# Granulocyte-Colony Stimulating Factor Improves Neurological and Functional Outcomes in Patients With Traumatic Incomplete Spinal Cord Injuries: A Systematic Review With Meta-Analyses

**DOI:** 10.1089/neur.2023.0099

**Published:** 2024-05-02

**Authors:** Luke J. Weisbrod, Thomas T. Nilles-Melchert, Judith R. Bergjord, Daniel L. Surdell

**Affiliations:** ^1^ Department of Neurosurgery, University of Nebraska Medical Center, Omaha, Nebraska, USA.; ^2^ Creighton University School of Medicine, Omaha, Nebraska, USA.

**Keywords:** granulocyte-colony stimulating factor, spinal cord injury, trauma

## Abstract

Spinal cord injury (SCI) is a cause for significant morbidity, often resulting in long-term disability. We compared outcomes after administration of granulocyte-colony stimulating factor (G-CSF) versus controls. MEDLINE, Embase, and Cochrane Library database searches yielded 222 records; six met study inclusion criteria. Fixed-effect and random-effects models were used to establish odds ratios (ORs) and mean difference (MD) with 95% confidence intervals (CIs) for each outcome. The results of the pooled analysis showed that in patients with incomplete SCI, G-CSF resulted in increased American Spinal Cord Injury Association (ASIA) motor scores at 3 months (MD = 0.57 [95% CI = 0.04, 1.10], *I*^2^ = 63.84%, *p* = 0.036), 6 months (MD = 4.18 [95% CI = 0.55, 7.80], *I*^2^ = 98.75%, *p* = 0.024), change in ASIA pinprick scores at 6 months (MD = 3.38 [95% CI = 1.48, 5.28], *I*^2^ = 89.78%, *p* < 0.001), and increased Spinal Cord Independence Measure (SCIM) III score at 6 months (MD = 3.27 [95% CI = 1.13, 5.41], *I*^2^ = 91.86%, *p* = 0.003). G-CSF resulted in more adverse events than the non-MP control groups (OR = 1.44 [95% CI = 0.38, 2.50], *I*^2^ = 0%, *p* = 0.008), but fewer than the MP control groups (OR = −4.2 [95% CI = −5.72, −2.68], *I*^2^ = 0%, *p* < 0.001). Systemic white blood cell count increased after administration of G-CSF in comparison to baseline (MD = 3.57 [95% CI = 2.79, 4.35], *I*^2^ = 55.06%, *p* < 0.001). G-CSF did not statistically increase ASIA Impairment Scale at 3 months (MD = 0.48 [95% CI = −0.33, 1.28], *I*^2^ = 0%, *p* = 0.246) or at 6 months (MD = 1.84 [95% CI = −0.10, 3.79], *I*^2^ = 50.09%, *p* = 0.063). These meta-analyses of six studies suggest that G-CSF for the treatment of incomplete SCI may result in improved neurological outcomes when compared to the controls. The results are limited by a small sample size with heterogeneity between studies. More robust prospective, randomized studies are necessary to help inform the safety and efficacy of G-CSF.

## Introduction

The National Spinal Cord Injury Statistical Center (NSCISC) has estimated the annual incidence of spinal cord injury (SCI) in the United States at ∼17,730 new cases annually, with ∼54 cases per million population.^1^ The prevalence of SCI in the United States has been estimated to be ∼291,000, or ∼881 per million population.^2,3^ The mean age at the time of injury is 33 years of age.^4^ SCI is accompanied by vast, prolonged social, physical, and monetary costs to persons and society, including the initial hospitalization, acute rehabilitation after discharge, modifications at home and to motor vehicles, and recurring costs for medications, supplies, and personal assistance. The extent of disability varies depending on the severity, level of injury, and whether additional injuries were present, as is common for polytrauma. On average, it has been estimated that a person with SCI will incur $1,130,000 in direct costs in the first year after the accident, followed by $196,107 annually thereafter.^5^

The pathophysiology of SCI is comprised of two sequential processes: primary and secondary injuries. Primary injury is the mechanical injury resulting from the trauma itself. Secondary injury is characterized by a cascade of biological reactions triggered by the primary injury and includes ischemia, hemorrhage, inflammation, oxidative stress, and apoptotic pathways, among others.^6^ The secondary injury is therefore the primary target for treatment of SCI and has been the focus of extensive research. By therapeutically attenuating the secondary injury, the spread of spinal cord parenchymal damage is suppressed, with the goal of improved neurological and functional prognoses.^7^

The acute management of SCI includes clinical assessment, characterization of the injury, immobilization with strict spinal precautions until instability has been excluded, critical care monitoring of hemodynamics and respiratory status with maintenance of mean arterial pressure >85 mm Hg in the first 5–7 days post-injury, and early surgical decompression and/or stabilization in appropriate cases.^8^ After immediate stabilization, measures are taken to prevent complications secondary to immobility, including deep venous thromboses, pneumonia, and pressure ulcers, among others. Rehabilitation during the inpatient admission and after discharge is essential to optimizing function in patients with SCI.^9^

Aside from the above core measures, there has not been a pharmacological therapeutic agent that has been shown to safely and effectively improve outcomes in patients who have sustained traumatic SCI. Historically, a treatment option was the early administration of high-dose methylprednisolone (MP). The mechanism of action of MP was to prevent secondary injury through anti-inflammatory effects and stabilization of the cell membrane.^10^ In the second National Acute Spinal Cord Injury Study (NASCIS-2) in 1990, a small beneficial effect of high-dose MP was reported for neurological recovery if administered within 8 h post-injury in patients with traumatic SCI.^10^ This conclusion, however, was drawn from *post hoc* subgroup analysis, which challenged the level of evidence.^11^ Recently, the inefficacy and significant potential for adverse effects and even death have been reported.^12,13^ As a result, the majority of up-to-date guidelines on the pharmacological therapy for acute SCI do not recommend steroids and its use is controversial.^14,15^ There is therefore a significant need for a novel drug that can attenuate secondary injury post-SCI without substantial associated adverse effects.

Granulocyte-colony stimulating factor (G-CSF) is a hematological cytokine that induces the development, proliferation, and survival of granulocyte lineage cells and in clinical practice is commonly used to treat neutropenia.^16^ Pre-clinical studies have shown that G-CSF promotes functional recovery in murine models of SCI through various mechanisms, including the mobilization of bone marrow cells into the injured spinal cord, suppression of inflammatory cytokines, suppression of neuron and oligodendrocyte apoptosis, and promotion of angiogenesis.^17–20^ Over the past couple of decades, the use of G-CSF for SCI has been investigated with retrospective and prospective clinical studies. The purpose of this systematic review and meta-analyses was to determine the effect of G-CSF in patients with traumatic SCI on neurological and functional outcome as well as its safety with respect to adverse events and mortality. Neurological outcomes of interest included change in the American Spinal Injury Association Scale (ASIA) motor and pinprick score and the change in ASIA Impairment Scale (AIS) grade. The functional outcomes of interest included changes to Spinal Cord Independence Measure III (SCIM III) scores.

## Methods

### Search strategy

A comprehensive, systemic literature search was conducted through the Cochrane Library, Embase, and MEDLINE databases on September 18, 2023 for articles investigating the utility of G-CSF compared to placebo or standard management of traumatic SCI following PRISMA (Preferred Reporting Items for Systematic Reviews and Meta-Analyses) guidelines.^21^ The search strategies included title and keywords that included the two search concepts: 1) granulocyte colony stimulating factor and 2) spinal cord injury. We focused the search on work with adult patients by first removing articles indexed as concerning animals given that these were not also indexed as concerning humans and then removing articles indexed as concerning pediatric age groups if these were not also indexed as concerning adults. Because funds were not available for translation, searches were limited to English-language articles, and because full study data were needed, conference abstracts, book chapters, and clinical trial registry records were excluded. The search yielded a total of 222 titles/abstracts for review.

Two independent researchers (L.W. and T.N.) screened the 222 articles identified by the search strategy. One hundred ninety-seven articles were excluded during the initial screen, and 25 articles were assessed for eligibility. Of these 25 articles, six were included for analysis and 19 were excluded. Reason for exclusion included lack of pre-specified data (10), duplicated patient population (five), administration of treatments in addition to G-CSF, inability to access data (one), and lack of comparison cohort (one).

### Selection criteria

We included all English-language articles that evaluated the administration of G-CSF for the treatment of traumatic SCI in comparison to placebo, no specific additional intervention, or MP. Criteria for inclusion in the study were: 1) traumatic etiology of SCI; 2) sample size of ≥5 patients in each group; 3) adult patient population with age ≥18 years; 4) available data regarding AIS, ASIA motor score, sensory recovery, functional outcome with SCIM III score, adverse events, change in systemic white blood cell (WBC) count, and mortality; and 5) human patients. Studies with non-extractable data and studies with overlapping patient data already included for the analysis were excluded.

### Outcomes

The outcomes of interest in this study included changes to AIS, ASIA motor score, ASIA pinprick score, SCIM III score, change in WBC after administration of G-CSF, adverse events, and mortality.

### Data extraction

Data were extracted independently by two researchers (L.W. and T.N.) and were collected using Microsoft Excel (Microsoft Corp., Redmond, WA). We recorded the following information: last name of the first author and year of study, country in which the study occurred, study dates, number of patients included in the study, age range, sex ratio, G-CSF dose, control group intervention, acuity of SCI, spinal cord level of injury, severity of SCI, whether surgery was performed, changes to AIS (defined as an increase in at least one grade), ASIA motor score and ASIA pinprick score at provided time points, change in WBC count before and after administration of G-CSF, adverse events, and mortality.

### Quality assessment

The Newcastle-Ottawa Scale was used to assess the quality of included studies that did not have a prospective, randomized design.^22^ Two reviewers (L.W. and T.N.) performed the quality assessments individually, and any discrepancies were resolved with discussion. Studies rated with 0–3 stars were considered low quality, studies with 4–6 stars were considered medium quality, and studies with 7–9 stars were considered high quality.

### Statistical analysis

Meta-analyses were performed to calculate pooled odds ratios (ORs) and mean difference (MD) with 95% confidence intervals (Cis) using a fixed-effect model for variables with low heterogeneity as measured by the *I*^2^ statistic and a random-effects model for continuous variables with higher risk for heterogeneity as measured by the *I*^2^ statistic. *I*^2^ values <25% were considered to have low heterogeneity, whereas all others were considered to have higher heterogeneity and were analyzed using the random-effects model. Statistical significance was achieved with a *p* value <0.05. Results are presented in forest plots. All analyses were completed using the meta-analysis functions in the open statistical software Jamovi version 2.4.7 (https://www.jamovi.org/).

## Results

### Search results

**FIG. 1.**
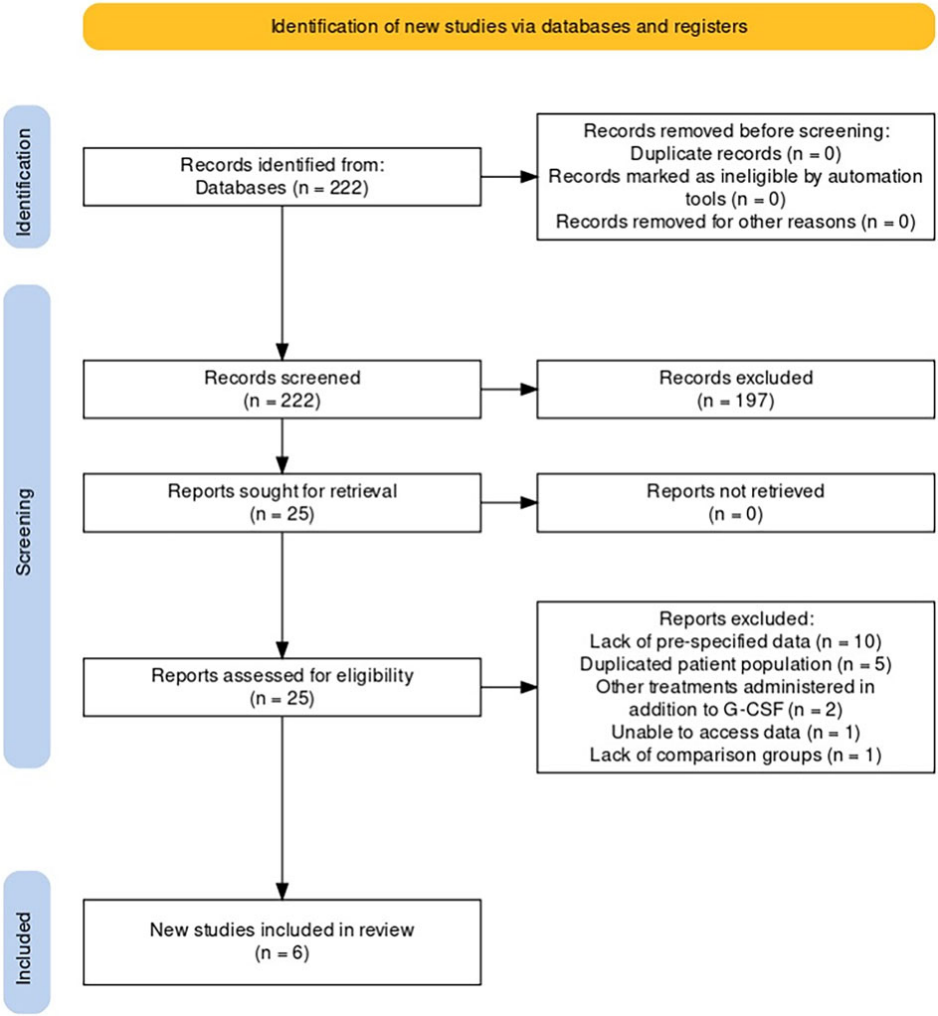
PRISMA (Preferred Reporting Items for Systematic Reviews and Meta-analyses) flowchart. G-CSF, granulocyte-colony stimulating factor.

MEDLINE, Embase, and Cochrane Library databases identified 222 publications (Fig. 1). In the final meta-analyses, six studies were included after studies that failed to meet inclusion criteria were removed. The characteristics of the six studies included in the meta-analyses are presented in Table 1.^23–28^ Four studies were performed in Japan, and two studies were performed in Iran. Three studies were prospective and randomized, two were prospective but non-randomized, and one was retrospective. The control intervention was normal saline subcutaneous placebo injection in three studies, high-dose MP per NASCIS II criteria in two studies as a historical control, and supportive cares without placebo intervention in one study.^10^ The dose and duration of G-CSF varied across studies and included 300 μg/day × 7 days in two studies, 400 μg/day in one study, 10 μg/day in one study, 5 μg/day × 5 days in one study, and a combination of 5 μg/kg/day × 5 days and 10 μg/day × 5 days among two separate patient populations in one study.

**Table 1.** Characteristics of Studies Included in Meta-Analyses

***Study***	***Country***	***Study design***	***Study dates***	***Newcastle-Ottawa Score***	***Control***	***Dose G-CSF (time from injury to admin)***	***Acuity***
Koda et al.^23^	Japan	PR	Not specified	NA	NS	400 μg/day × 5 days (<48 h)	<48 h
Derakhshanrad et al.^24^	Iran	PR	Aug 2014 to Feb 2016	NA	NS	300 μg/day × 7 days (1–6 months)	1–6 months
Derakhshanrad et al.^25^	Iran	PR	Jun 2013 to Jun 2016	NA	NS	300 μg/day × 7 days (6 months)	6 months
Kamiya et al.^26^	Japan	R	Aug 2009 to Jul 2012	6	MP	10 μg/kg/day × 5 days (<48 h)	<48 h
Inada et al.^27^	Japan	PNR	Aug 2009 to Mar 2011	7	None	5 μg/kg/day × 5 days (<48 h)	<48 h
Takahashi et al.^28^	Japan	PNR	Apr 2008 to Mar 2010	6	MP	5 μg/kg/day × 5 days; 10 μg/kg/day × 5 days (<48 h)	<48 h

R, retrospective; PR, prospective randomized; PNR, prospective non-randomized; NA, not applicable; NS, normal saline; MP, methylprednisolone; G-CSF, granulocyte colony stimulating factor; admin, administration.

Regarding acuity of injury, four studies were within the acute period post-SCI defined as <48 h from time of injury at the time of intervention, one study was within the subacute period from 1 to 6 months post-injury, and one study was within the chronic period at 6 months post-injury. The timing from injury to administration of G-CSF was <48 h in four studies, between 1 and 6 months in one study, and 6 months in one study.

The meta-analyses included 360 patients whose demographics are included in Table 2. Of the 360 total patients, 170 received G-CSF. The control groups included a total of 190 patients, 129 patients who received a normal saline subcutaneous placebo injection, 42 patients who received high-dose MP, and 19 patients who received supportive cares without a specific placebo intervention (Table 2). The age range of the G-CSF and control groups was 23–78 and 18–85 years, respectively. An elevated male-to-female ratio was observed in all G-CSF and control groups aside from one control group where the ratio was 0.88. The range of male-to-female ratios of the G-CSF and control groups ranged from 1.83–8.33 and 0.88–12, respectively. In three studies, the level of SCI was cervical spine only; in the remaining three studies, the level of SCI included cervical, thoracic, and lumbar spine. In four studies, the severity of SCI was incomplete, defined as residual motor or sensory function more than three segments below the level of injury.^29^

**Table 2.** Characteristics of Patient Demographics Included in Meta-Analyses

***Study***	***N G-CSF***	***N Cl***	***Age range G-CSF***	***Age range Cl***	***Sex ratio M:F G-CSF***	***Sex ratio M:F Cl***	***Level***	***Severity***	***Surgery?***
Koda et al.^23^	43	45	51–78	52–78	5.14	0.88	C	I	NS
Derakhshanrad et al.^24^	28	26	23–50	21–41	8.33	12	C/T/L	I	Yes
Derakhshanrad et al.^25^	56	58	24–46	26–48	7	8.67	C/T/L	I	Yes
Kamiya et al.^26^	12	14	38–72	18–85	3:1	3.86	C	I	NS
Inada et al.^27^	16	19	38–68	23–85	1.83	3.8	C	I/Co^a^	Yes
Takahashi et al.^28^	15	28	38–68	18–75	4.33	4.6	C/T	I/Co^a^	NS

^a^ Complete spinal cord injuries not included in analyses.

N, number; Cl, control; M, male; F, female; C, cervical; T, thoracic; L, lumbar; I, incomplete spinal cord injury; Co, complete spinal cord injury; G-CSF, granulocyte-colony stimulating factor; NS, not specified.

Two studies included both incomplete and complete SCI. Of these two studies, only patients with incomplete SCI were included in meta-analyses because there was only 1 patient in each study who received G-CSF with a SCI. Three studies commented that surgical decompression and/or fixation was performed if indicated based on surgeon discretion during a therapeutic window that they deemed acceptable. The remaining three studies did not mention whether surgical intervention was performed.

The adverse events and mortality of patients included in the meta-analyses are included in Table 3. There were zero mortalities or severe adverse events reported in the G-CSF or control groups. There was a total of 27 adverse events reported of the 170 patients who received administration of G-CSF; 6 patients had fever, 5 patients had bone pain, 5 patients had a urinary tract infection (UTI), in 2 patients the adverse event was not specified, 1 patient had muscle pain, 1 patient had headache, 1 patient had transient itching/skin rash, 1 patient had left upper quadrant pain (LUQ), 1 patient had pneumonia, 1 patient had transient hepatic dysfunction, and 1 patient had hepatopathy. In the MP group, 39 of 42 patients had reported adverse events, whereas in the non-MP control groups, 4 of 148 patients had reported adverse events.

**Table 3.** Adverse Events and Mortality of Patients Included in Meta-Analyses

	***G-CSF 5 μg/kg/day × 5 days***	***G-CSF 10 μg/kg/day × 5 days***	***G-CSF 300 μg/day × 7 days***	***G-CSF 400 μg/day × 5 days***	***MP***	***Control***	***Total***
Patients, ***N***	21	22	84	43	42	148	360
Patients w/ any AE	2	6	14	5	39	4	70
Patients w/ severe AE	0	0	0	0	0	0	0
Mortality	0	0	0	0	0	0	0
Fever			1	5			6
Bone pain			5				5
UTI	1	4			17		22
Nausea/vomiting			2			1	3
Muscle pain			1				1
Headache			1			1	2
Transient itching/skin rash			1				1
LUQ pain			1			1	2
Pneumonia		1			16		17
Transient hepatic dysfunction	1						1
Hepatopathy		1			1		2
Gastric ulcer					5		5
Not specified respiratory						3	3
Not specified			2				2

*N*, number; AE, adverse event; UTI, urinary tract infection; LUQ, left upper quadrant; G-CSF, granulocyte-colony stimulating factor; MP, methylprednisolone

### Meta-analyses

#### American Spinal Cord Injury Association Impairment Scale 3 months

**FIG. 2.**
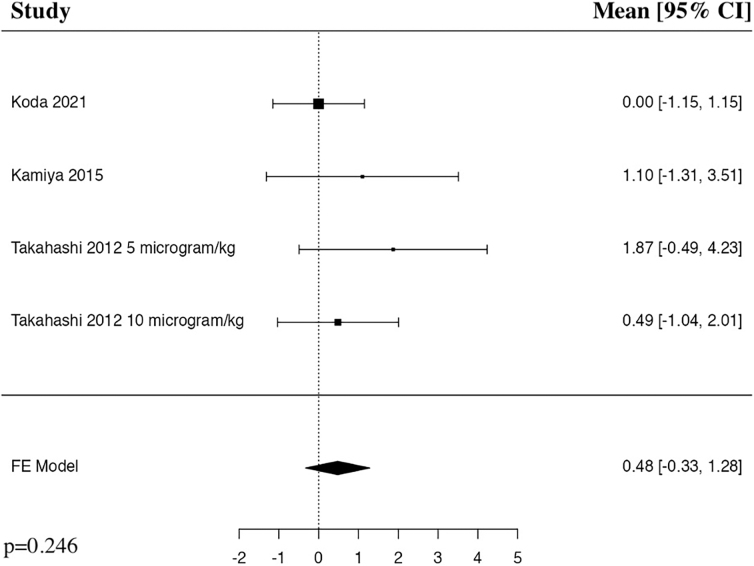
Forest plot demonstrating a fixed-effects model for MD of AIS at 3 months in patients with traumatic SCI who received G-CSF in comparison to control groups.^23,26,28^ AIS, American Spinal Cord Injury Association Impairment Scale; CI, confidence interval; FE, fixed-effects; G-CSF, granulocyte-colony stimulating factor; MD, mean difference.

The meta-analysis of change in AIS at 3 months included three studies and four patient populations who received G-CSF. The results of the pooled analysis showed a trend toward an increased change in AIS at 3 months in the G-CSF group compared to control groups, but was not statistically significant (MD = 0.48, 95% CI [–0.33, 1.28], *I^2^* = 0%, *p* = 0.246; Fig. 2).

#### American Spinal Cord Injury Association Impairment Scale 6 months

**FIG. 3.**
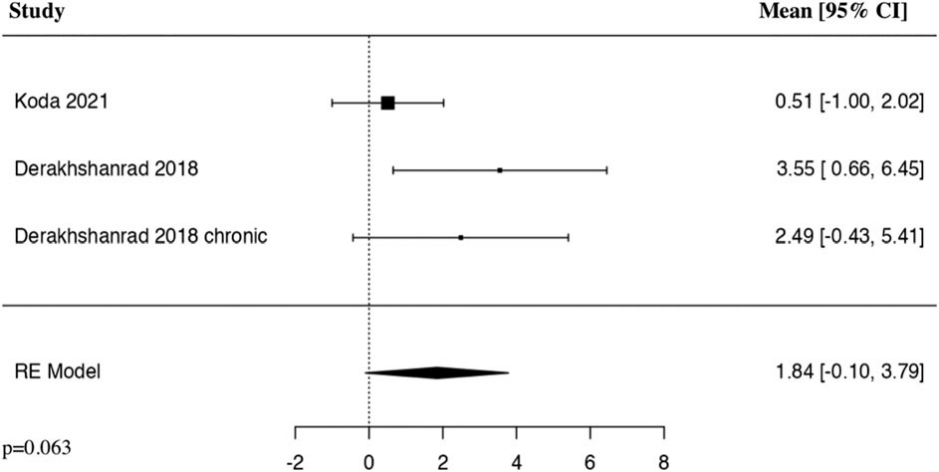
Forest plot demonstrating random-effects model for MD of AIS at 6 months in patients with traumatic SCI who received G-CSF in comparison to control groups.^23–25^ AIS, American Spinal Cord Injury Association Impairment Scale; CI, confidence interval; G-CSF, granulocyte-colony stimulating factor; MD, mean difference; RE, random-effects; SCI, spinal cord injury.

The meta-analysis of change in AIS at 6 months included three studies. The results of the pooled analysis showed a further trend toward an increase in the change in AIS at 6 months in the G-CSF group compared to control groups, but was not statistically significant (MD = 1.84, 95% CI [–0.10, 3.79], *I^2^* = 50.09%, *p* = 0.063; Fig. 3).

#### American Spinal Cord Injury Association motor 3 months

**FIG. 4.**
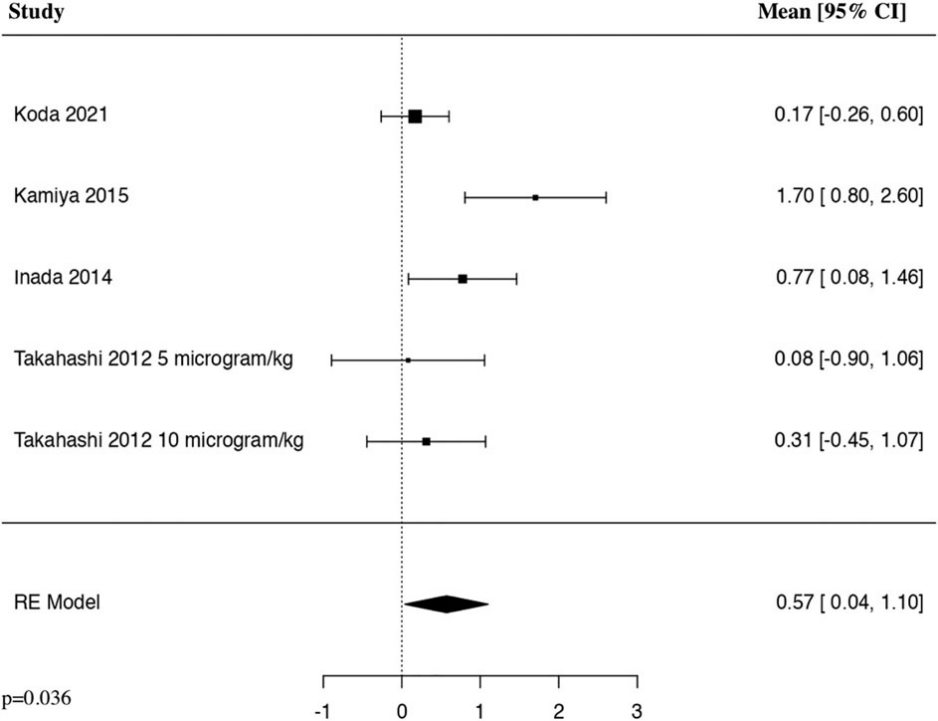
Forest plot demonstrating a random-effects model for MD of change in ASIA motor score at 3 months in patients with traumatic SCI who received G-CSF in comparison to control groups.^23,26–28^ ASIA, American Spinal Cord Injury Association; CI, confidence interval; G-CSF, granulocyte-colony stimulating factor; MD, mean difference; RE, random effects; SCI, spinal cord injury.

The meta-analysis of change in ASIA motor score at 3 months included four studies and five patient populations who received G-CSF. The results of the pooled analysis showed that the administration of G-CSF resulted in a statistically significant increase in the change in ASIA motor score at 3 months in comparison to control groups in patients with traumatic SCI (MD = 0.57, 95% CI [0.04, 1.10], *I^2^* = 63.84%, *p* = 0.036; Fig. 4).

#### American Spinal Cord Injury Association motor 6 months

**FIG. 5.**
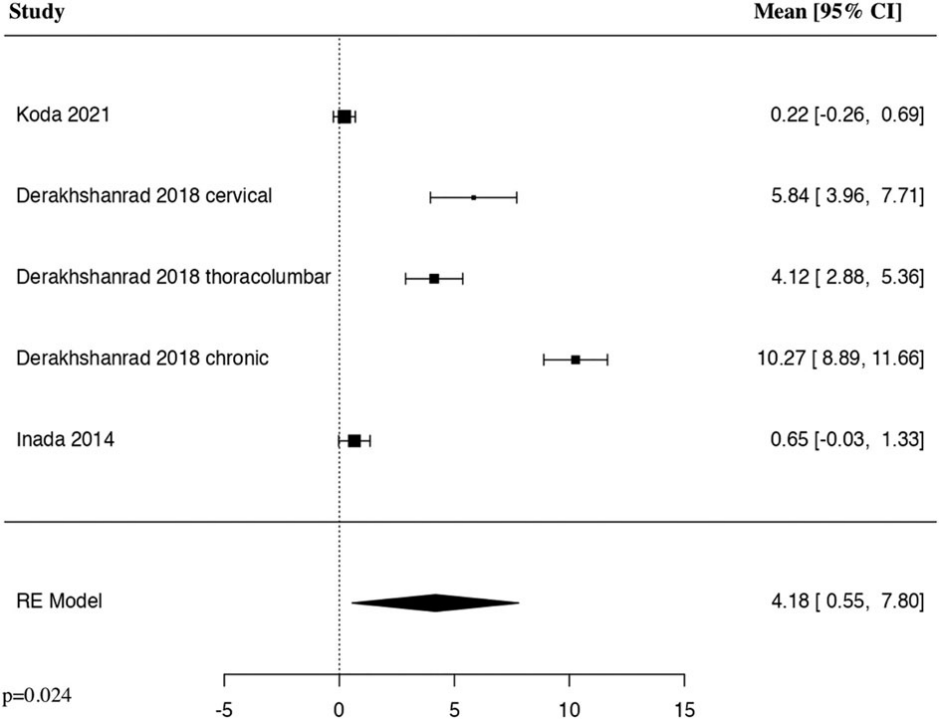
Forest plot demonstrating random-effects model for MD of change in ASIA motor score at 6 months in patients with traumatic SCI who received G-CSF in comparison to control groups.^23–25,27^ ASIA, American Spinal Cord Injury Association; CI, confidence interval; G-CSF, granulocyte-colony stimulating factor; MD, mean difference; RE, random effects; SCI, spinal cord injury.

The meta-analysis of change in ASIA motor score at 6 months included four studies and five patient populations who received G-CSF. The results of the pooled analysis showed that the administration of G-CSF resulted in a statistically significant increase in the change in ASIA motor score at 6 months in comparison to control groups (MD = 4.18, 95% CI [0.55, 7.80], *I^2^* = 98.75%, *p* = 0.024; Fig. 5).

#### American Spinal Cord Injury Association pinprick 6 months

**FIG. 6.**
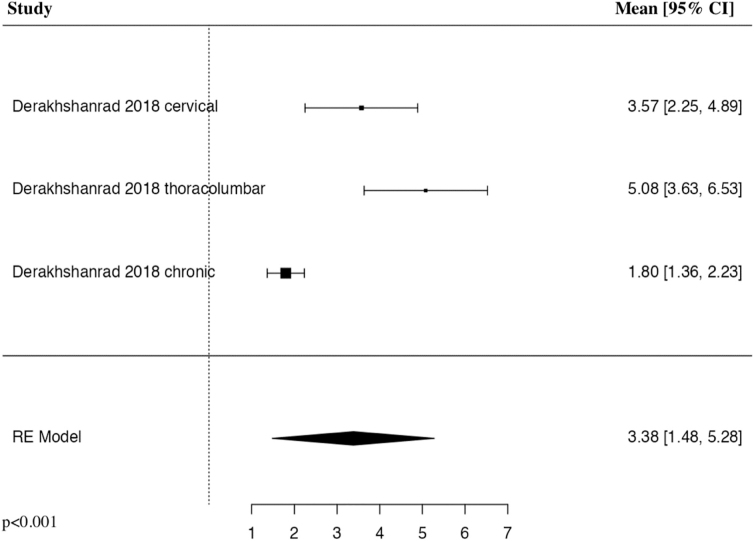
Forest plot demonstrating random-effects model for MD of change in ASIA pinprick score at 6 months in patients with traumatic SCI who received G-CSF in comparison to control groups.^24,25^ ASIA, American Spinal Cord Injury Association; CI, confidence interval; G-CSF, granulocyte-colony stimulating factor; MD, mean difference; RE, random effects; SCI, spinal cord injury.

The meta-analysis of change in ASIA pinprick score at 6 months included two studies and three patient populations who received G-CSF. The results of the pooled analysis showed that the administration of G-CSF resulted in a statistically significant increase in the change in ASIA pinprick score at 6 months in comparison to control groups (MD = 3.38, 95% CI [1.48, 5.28], *I^2^* = 89.78%, *p* < 0.001; Fig. 6).

#### Spinal Cord Independence Measure III 6 months

**FIG. 7.**
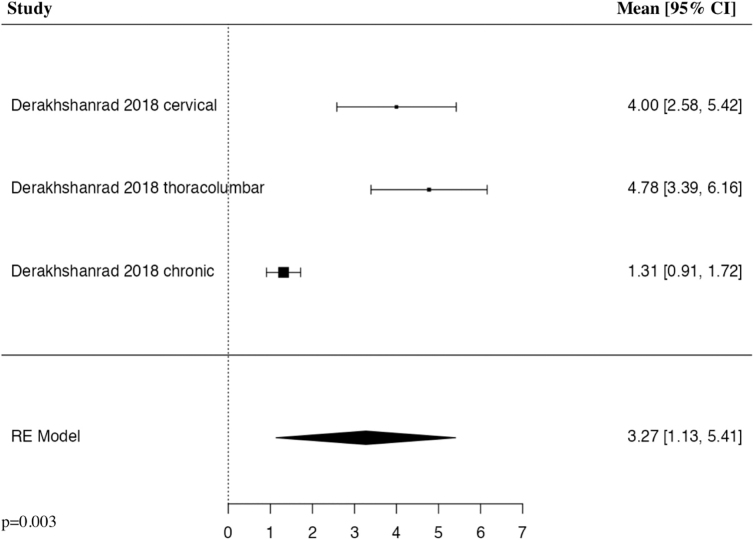
Forest plot demonstrating random-effects model for MD of change in SCIM III score at 6 months in patients with traumatic SCI who received G-CSF in comparison to control groups.^24,25^ CI, confidence interval; G-CSF, granulocyte-colony stimulating factor; MD, mean difference; RE, random effects; SCI, spinal cord injury; SCIM, Spinal Cord Independence Measure.

The meta-analysis of change in SCIM III score at 6 months included two studies and three patient populations who received G-CSF. The results of the pooled analysis showed that the administration of G-CSF resulted in a statistically significant increase in the change in SCIM III score at 6 months in comparison to control groups in patients with traumatic SCI (MD = 3.27, 95% CI [1.13, 5.41], *I^2^* = 91.86%, *p* = 0.003; Fig. 7).

#### Change in white blood cell count

**FIG. 8.**
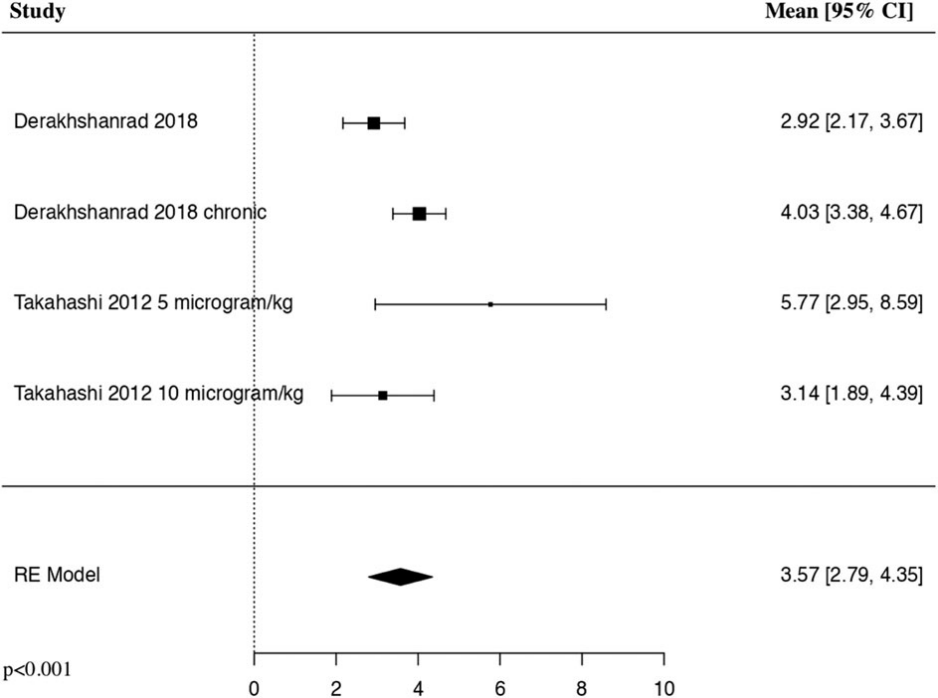
Forest plot demonstrating random-effects model for MD of median WBC count after G-CSF in comparison to before administration in patients with traumatic SCI.^24,25,28^ CI, confidence interval; G-CSF, granulocyte-colony stimulating factor; MD, mean difference; RE, random effects; SCI, spinal cord injury; WBC, white blood cell

The meta-analysis of change in systemic WBC counts after G-CSF administration in comparison to before administration in patients with traumatic SCI included three studies and four patient populations who received G-CSF. The results of the pooled analysis showed that the administration of G-CSF resulted in a statistically significant increase in the systemic WBC count in comparison to before administration in patients with traumatic SCI (MD = 3.57, 95% CI [2.79, 4.35], *I^2^* = 55.06%, *p* < 0.001; Fig. 8).

### Adverse events

**FIG. 9.**
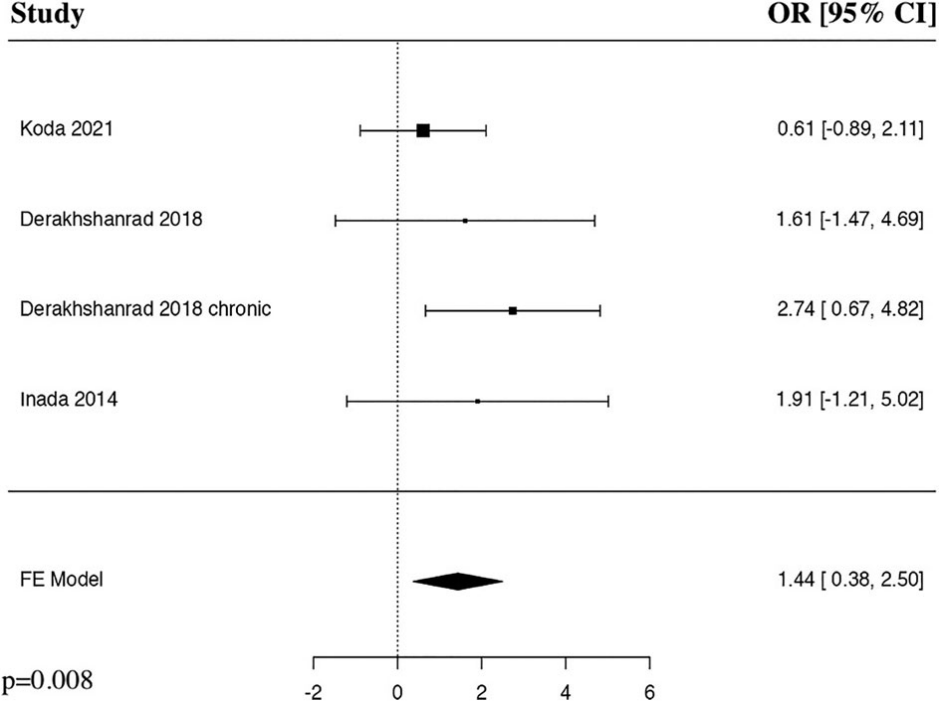
Forest plot demonstrating fixed-effects model for adverse events in patients who received G-CSF in comparison to non-MP control groups for traumatic SCI.^23–25,27^ CI, confidence interval; FE, fixed-effects; G-CSF, granulocyte-colony stimulating factor; MP, methylprednisolone; OR, odds ratio; SCI, spinal cord injury

The meta-analysis of adverse events after G-CSF in comparison to non-MP control included four studies. The results of the pooled analysis showed that the administration of G-CSF resulted in a statistically significant increase in adverse events in comparison to non-MP control groups in patients with traumatic SCI (OR = 1.44, 95% CI [0.38, 2.50], *I^2^* = 0%, *p* = 0.008; Fig. 9).

**FIG. 10.**
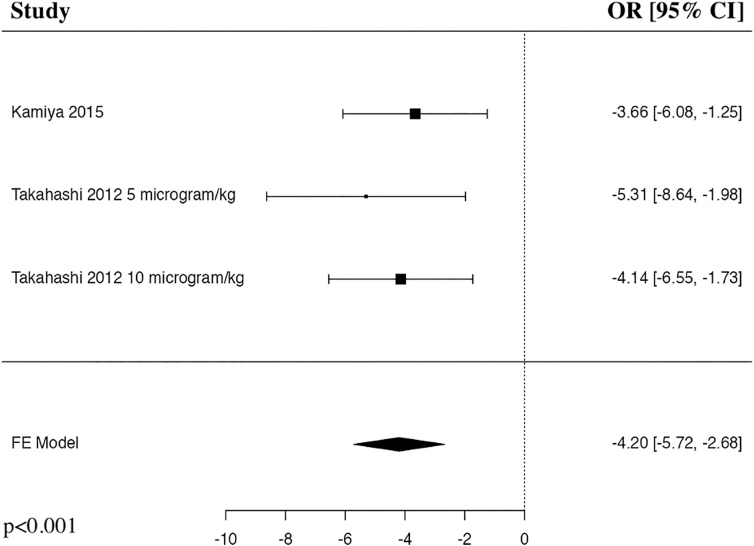
Forest plot demonstrating fixed-effects model for adverse events in patients who received G-CSF in comparison to MP for traumatic SCI.^26,28^ CI, confidence interval; FE, fixed-effects; G-CSF, granulocyte-colony stimulating factor; MP, methylprednisolone; OR, odds ratio; SCI, spinal cord injury.

The meta-analysis of adverse events after G-CSF in comparison to MP control included two studies and three patient populations who received G-CSF. The results of the pooled analysis showed that the administration of G-CSF resulted in a statistically significant decrease in adverse events in comparison to the MP control in patients with traumatic SCI (OR = −4.2, 95% CI [–5.72, −2.68], *I^2^* = 0%, *p* < 0.001; Fig. 10).

## Discussion

In experimental studies on acute myocardial infarction, it has been shown that G-CSF mobilizes stem cells into the myocardium, as a result protecting cardiac tissue from further injury.^30^ In neurological models, such as for ischemic stroke, G-CSF has been shown to protect the brain from further injury by reducing the expression of inflammatory cytokines and suppressing neuronal apoptosis.^31–33^ Similar observations have been made in pre-clinical murine models of acute SCI.^20–22^ Given these results, clinical trials have been initiated for acute myocardial infarction, as well as neurological disorders including cerebral infarction and amyotrophic lateral sclerosis.^34–36^ These studies reported the safety and feasibility of G-CSF administration^39^ as well as improved neurological symptoms.^34–36^ This systematic review includes meta-analyses of the current clinical studies investigating G-CSF for the treatment of traumatic SCI.

The results of our meta-analyses show that there were no severe adverse effects related to G-CSF, nor were there any mortalities. The results presented did suggest that G-CSF results in an overall increase in adverse events in comparison to control groups, many of which were transient, constitutional, and non-specific in nature. The adverse event profile for G-CSF was, however, significantly less in comparison to high-dose MP, a pharmacological agent commonly used historically in the treatment of SCI, which has become controversial yet is still routinely observed in practice.

Given that G-CSF stimulates the bone marrow, an observed effect is an increase in systemic WBC count after administration, which was consistent with our results that demonstrated a statistically significant increase in systemic WBC count after administration. A couple of study protocols included in the meta-analyses noted that G-CSF administration was to be stopped if WBC >50,000 cells/mm^3^ and if platelets <100,000 cells/mm^3^ because there is an increased risk of splenic rupture if WBC >50,000 cells/mm^3^.^2,25,26,29^ Of the mentioned studies, only 1 patient who received a dose of 10 μg/kg/day developed a WBC increase by >50,000 cells/mm^3^ that returned to pre-administration levels 1 day after the end of administration and did not result in splenic rupture or other severe adverse events.^28^ Consistent with previous reports, our results would suggest that G-CSF is an overall safe and feasible pharmacological option with a mild-to-moderate, often transient, side-effect profile.

The results of our meta-analyses show a statistically significant improvement in functional outcomes with SCIM III at 6 months, as well as neurological outcome with regard to change in ASIA motor score at 3 and 6 months and change in ASIA pinprick score at 6 months in comparison to controls. A significant increase in AIS at 3 months after administration of G-CSF was not shown on meta-analysis and in only a prospective non-randomized study was a significant increase shown at 3 months.^27^ Most previously reported clinical trials for SCI use 6 months post-injury as the time point for evaluating primary end-points.^10,37,38^ Given that neurological injury can occur 3–12 months after SCI, 3 months may be too early to detect significant change given that there was a greater increase in AIS at 6 months on meta-analysis, but still not quite to a level of statistical significance. Although the meta-analyses show statistically significant changes in SCIM III as well as ASIA motor and pinprick scores, it is possible that the effect of G-CSF is just marginal enough to not significantly result in an observed full grade increase in AIS in comparison to controls.

Overall, the results of this study suggest that G-CSF may be a reasonably safe and appropriate pharmacological treatment option to optimize neurological and functional outcomes in patients with traumatic incomplete SCI. However, these results should prompt further study into the efficacy of G-CSF. Large, randomized trials should be performed to establish the benefit G-CSF compared to placebo for the management of traumatic SCI, and specific attention should be given to parse out the ideal dose, duration, and time frame for administration.

### Limitations

This study is limited by the pooled data available from the relatively small series included in the analyses, many of which were retro- or prospective, but non-randomized. Two of the studies included in the meta-analyses were performed by the same group of authors, which is a potential source of bias. Among the studies, there was significant heterogeneity among the study protocols with no standardized dose or duration of the administered G-CSF, acuity of SCI, or control group intervention, nor was there a standardized approach or time frame for surgical decompression and/or fixation.

In future study protocols, the ideal measure of G-CSF's efficacy in SCI would be to compare its efficacy with that of a placebo in a randomized, prospective, and blinded fashion. Patients would ideally not receive high-dose MP, given that this is a confounding intervention. Given high-dose MP's history as the initial treatment option for SCI and its small beneficial effect, it has taken time for it to fall out of practice patterns and is still commonly administered despite its reported adverse effects. Our study is limited in that two of the studies included for pooled analysis were from over a decade ago when high-dose MP was even more common in practice and studies commonly included patient populations who received high-dose MP.

Whereas the majority of studies included for meta-analyses were within the acute period <48 h after traumatic SCI, two of the studies included patient populations in which the administration of G-CSF spanned as many as 6 months post-injury. We chose to include studies ranging out to 6 months from the time of injury with the knowledge that G-CSF may lose effectiveness the further out from injury that it is administered and highlighting these outlier study populations in the pooled analysis. In the future, when larger sample sizes are present, it would be of interest to subanalyze based on SCI acuity to parse out the ideal timing of administration of G-CSF for its maximal safety and effectiveness.

In regard to demographics, many of the studies were represented by a high male-to-female ratio, even after accounting for a male predilection for traumatic SCI in males that is typically 3–4 times higher than females.^39^ It is worth noting that the studies included for meta-analyses occurred in Japan and Iran, where there are differences present among the patient population and healthcare systems compared to those in Western practice. Therefore, the meta-analyses may not be generalizable to a diverse population. Further, only patients with incomplete SCI were included for meta-analyses. There may be differences in response to G-CSF between these groups, which has been demonstrated in one prospective non-randomized study in which patients with incomplete SCI had significantly more improvement in change in ASIA motor score when compared to motor-complete patients, as well as significant improvement in light touch and pinprick scores.^40^

Finally, there was significant heterogeneity among the studies included for meta-analyses, particularly for the change in WBC, SCIM III at 6 months, ASIA pinprick at 6 months, ASIA motor at 3 months, and AIS at 6 months. Therefore, the results of this analysis should be interpreted with caution until more robust prospective randomized clinical trials are performed to help inform the utility of G-CSF versus placebo control.

## Conclusion

The results of these meta-analyses suggest that G-CSF for the treatment of traumatic incomplete SCI results in improved neurological outcomes with respect to change in ASIA motor score at 3 and 6 months, change in ASIA pinprick score at 6 months, and improved functional outcome with improved SCIM III score at 6 months when compared to normal saline placebo, supportive cares without specific placebo intervention, or high-dose MP. G-CSF was associated with an increase in adverse events in comparison to normal saline placebo, but less adverse events when compared with high-dose MP. G-CSF resulted in an increase in systemic WBC count from baseline, but was not associated with severe adverse events or mortality. More robust prospective randomized studies are necessary to help inform the safety and efficacy of G-CSF for traumatic SCI.

## Transparency, Rigor, and Reproducibility Summary

The study design and analysis plan were pre-registered on September 19, 2023 at PROSPERO, the international prospective register of systematic reviews (https://www.crd.york.ac.uk/prospero/) registration identification CRD42023465221. The aim of this study was to assess the safety and efficacy of G-CSF in treating traumatic SCI. Primary outcomes to be evaluated were changes to ASIA motor score, ASIA pinprick score, AIS, SCIM III score, change in systemic WBC count after administration of G-CSF, adverse events, and mortality. A professional librarian conducted a comprehensive, systematic literature review in the Cochrane Library, Embase, and MEDLINE databases on September 18, 2023 in accordance with PRISMA guidelines. Search strategies were conducted to identify all articles pertaining to G-CSF and SCI. Initial screening of the 222 identified articles yielded 25 articles for further assessment. Six articles met inclusion criteria upon further assessment. There were no disagreements between reviewers.

Data were extracted manually from the included articles; all data were recorded on a Microsoft Excel file. The quality of the included studies was evaluated independently by two reviewers. Discrepancies were resolved by discussion and common consensus. Open statistical software (Jamovi 2.4.7) was used for statistical analysis. *I*^2^ values were calculated to determine heterogeneity; *I*^2^ <25% was considered low heterogeneity whereas >25% was considered high heterogeneity. Fixed-effect and random-effects estimation models were used depending on level of heterogeneity; fixed-effect model for low heterogeneity and random-effects model for high heterogeneity. The meta-analyses are reproducible. Database search terms, search results, and extracted data are available on request. This study followed the PRISMA checklist, and the PRISMA flowchart is included. This study did not involve any identifiable patient data; institutional review board approval was not necessary. The authors report no conflicts of interest, and this study did not receive funding to conduct. This study is susceptible to publication bias. Limitations of this study are a low number of included studies and a couple of the studies conducted by the same group of authors.

## Abbreviations Used

AIS: ASIA Impairment Scale
ASIA: American Spinal Cord Injury Association
CI: confidence interval
G-CSF: granulocyte-colony stimulating factor
LUQ: left upper quadrant
MD: mean difference
MP: methylprednisolone
OR: odds ratio
SCI: spinal cord injury
SCIM: Spinal Cord Independence Measure
UTI: urinary tract infection
WBC: white blood cell count
